# The serostatus of *Brucella* spp., *Chlamydia abortus*, *Coxiella burnetii* and *Neospora caninum* in cattle in three cantons in Bosnia and Herzegovina

**DOI:** 10.1186/s12917-018-1361-z

**Published:** 2018-02-02

**Authors:** Adis Softic, Kassahun Asmare, Erik Georg Granquist, Jacques Godfroid, Nihad Fejzic, Eystein Skjerve

**Affiliations:** 1 Department for economics and animal health, University of Sarajevo, Veterinary Faculty in Sarajevo, Zmaja od Bosne 90, 71000 Sarajevo, Bosnia and Herzegovina; 2 Hawassa University School of Veterinary Medicine, P.O. Box 5, Hawassa, Ethiopia; 3 Norwegian University of Life Sciences, Faculty of Veterinary Medicine, P.O. Box 8146, 0033 Oslo, Norway; 4 University in Tromsø, Faculty of Biosciences, Fisheries and Economics, Postboks 6050 Langnes, 9010 Tromsø, Norway

**Keywords:** *C. abortus*, *C. Burnetii*, *N. Caninum*, *Brucella* spp., Cattle, Bosnia and Herzegovina

## Abstract

**Background:**

Dairy production in Bosnia and Herzegovina exhibits limited productivity, which may partly, be explained by extensive reproductive problems of non-infectious and infectious origin. *Brucella* spp., *Chlamydia abortus*, *Coxiella burnetii* and *Neospora caninum* are common infectious causes of decreased reproductive outcomes in cattle worldwide. Little is, however, known about the disease status of herds with reduced reproductive performances. A cross-sectional study was designed to document the status of these pathogens in dairy cattle in Bosnia and Herzegovina. A total of 1970 serum samples were collected from cattle in farms located in three cantons (regions). Enzyme linked immunosorbent assays were used to screen for seropositivity against four selected pathogens.

**Results:**

The overall seroprevalence was estimated at both the herd level and at individual level for each pathogen. At the individual animal level, the prevalence for *C. abortus, C. burnetii*, *N. caninum* and *Brucella* spp. was 52.1% (95% CI: 41.2–62.7), 8.8% (95% CI: 5.3–14.2), 9.2% (95% CI: 6.0–12.3 and 0.2% (95% CI: 0.1–0.5), respectively. The corresponding estimates for herd level were 87.9% (95% CI: 82.6–91.8), 19.6% (95% CI: 14.6–25.8), 35.2% (95% CI: 28.8–42.1), and 1.5% (95% CI: 0.5–4.6). A substantial overlap was observed in the presence of *N. caninum*, *C. abortus* and *C. burnetii* at individual and herd level.

**Conclusion:**

Our study demonstrated a high level of antibodies to *Chlamydia abortus*. Considering the association of this agent with reproductive disorders in cattle, future studies should be directed to the epidemiological traits of this infection. Additionally, the relatively high levels of exposure to *C. burnetii* and *N. caninum* found in this study highlights the need for targeted control of infectious causes of reproductive disorders in dairy cattle of the studied areas. Given the low seroprevalence, *Brucella* spp. does not seem to represent a problem in the reproductive health of cattle in the studied areas.

**Electronic supplementary material:**

The online version of this article (10.1186/s12917-018-1361-z) contains supplementary material, which is available to authorized users.

## Background

The cattle industry is faced with a number of challenges affecting its further development. Reproductive disorders represent one of these challenges across the world, and may be due to intrinsic or extrinsic factors imposed on the herd and individual animals such as genotypic traits, feeding, contaminants and toxins in feeds or other environmental factors [1]. The incidences of reproductive diseases in cattle are reported to be increasing over the years [1]. Infectious agents are known to cause infertility, early embryonic death, protracted calving seasons, abortion and stillbirth [2, 3]. These infectious agents could seriously damage general agriculture of countries in transition, such as Bosnia and Herzegovina.

Agriculture has been an indispensable part of the gross economy in Bosnia and Herzegovina (BH) over the last decade. Growing market demands and convergence to standards set by the European Union forces animal husbandry practices to shift from extensive to semi-intensive production system, along with improved biosecurity and focus on herd health status [4]. In the wake of structural changes, the enhancement of reproductive performance of livestock is a necessity in contributing to increased production [5].

However, little is known about the epidemiology of various infectious agents in the cattle populations of BH. Several viral, parasitic or bacterial pathogens are known to be associated with reproductive failure in cattle, including bovine viral diarrhoea virus (BVDV), infectious bovine rhinotracheitis (IBR) virus, *Brucella abortus*, occasionally *B. melitensis*, *Neospora caninum*, *Coxiella burnetii*, *Campylobacter fetus venerealis* or *C. fetus fetus*, *Leptospira* spp., *Tritrichomonas foetus*, *Chlamydia abortus* [6–10]. Some of these infectious agents are ubiquitous in cattle populations, and their occurrence is associated with biosecurity measures in farms. However, in-herd disease control measures are rarely effective, since viruses and bacteria may be shed continuously through faecal, vaginal, urine, seminal, ocular, and nasal discharges. Vertical transmission also occurs frequently with BVDV, *Brucella* spp., *Campylobacter* spp. and *N. caninum* [7–10].

Control of reproductive disorders in cattle relies upon systematic and coordinated efforts of the country’s Veterinary Services at the national and regional levels and requires financial resources. Trade and transportation of animals are contributing factors in the spread of diseases. Hence, controlling herd biosecurity and adopting artificial insemination may aid in the prevention of contagious reproductive diseases. Until now, there has been very limited information on the occurrence of infectious reproductive problems in BH. In this study, the authors focused on four of the assumed important agents: *Brucella* spp., *C. abortus*, *C. burnetii*, and *N. caninum.* The selection was based upon the relative importance of these agents in the occurrence of reproductive problems in cattle worldwide. Additionally, the limited information about the importance of *Brucella* spp. and *C. burnetii* and the uncertainty about the presence of *C. abortus* and *N. caninum* in cattle in BH contributed to the selection.

**Chlamydiae** are obligate intracellular, Gram-negative bacteria that cause a wide range of diseases in animals and humans [11]. Some of the chlamydiae are ubiquitous in cattle populations. Infections of cattle with *Chlamydia abortus*, *C. pecorum, C. psittaci and Chlamydia suis* have been associated with reproductive disorders including abortion, endometritis, repeat breeding, vaginitis, birth of weak calves and perinatal mortality. Chlamydial diseases are frequently asymptomatic in nature, and clinical expressions among individual cattle are often noticed as a non-specific loss in reproduction. Infection with chlamydiae has also been associated with bovine sub-clinical mastitis. Also, clinical manifestation in calves are recorded as pneumonia and weight loss [3, 11–13].

***C. burnetii*** is a rickettsial pathogen which causes Q-fever in cattle [14]. The infection is generally asymptomatic but can lead to abortions, premature offspring, stillbirths and delivery of weak offspring [6]. Worldwide, the apparent prevalence is slightly higher in cattle than in small ruminants [15].

**Brucellosis** (*Brucella* spp. infection) in cattle may result in abortion after the fifth month of pregnancy or delivery of weak calves. In addition, retained fetal membrane and metritis often occurs [2, 12, 16]. Cows with puerperal metritis resulting from retained placenta are prone to uterine diseases such as clinical metritis, clinical endometritis and subclinical endometritis, which lead to failure of conceiving. When a successful eradication of *B. abortus* has been implemented in cattle, sporadic infection of cattle with *B. melitensis* have been documented in Spain [17] and France [18], countries that are not free of brucellosis in small ruminants. *B. melitensis* biovar 3 is the only *Brucella* spp. isolated from cattle, small ruminants and humans in BH [19, 20], and *B. abortus* has never been isolated. However, little effort has been put into epidemiological mapping of disease outbreaks and phylogenetic studies on pathogens associated with livestock in BH. The implementation of the national brucellosis mass vaccination program in sheep and goats since July 2009 (with ocular Rev-1 vaccine) has resulted in the successful control of brucellosis in small ruminants with prevalence rates steadily decreasing [21].

***N. caninum*** is a protozoan parasite of cattle. Dogs are definitive hosts while cattle are intermediate hosts. Transmission is by two ways: highly effective trans-placental (vertical) or post-natal (horizontal) via the oocysts ingestion from feces of dogs. [22, 23]. Cows of any age may abort from 3 month of gestation to term, but most abortions occur at 5–6 month of gestation [22, 24].

To better understand the epidemiological pattern of these infections, a cross-sectional serological study was designed to describe the epidemiological characteristics of *Brucella* spp., *C. abortus*, *C. burnetii* and *N. caninum* in BH.

## Methods

### Study area

**Fig. 1:**
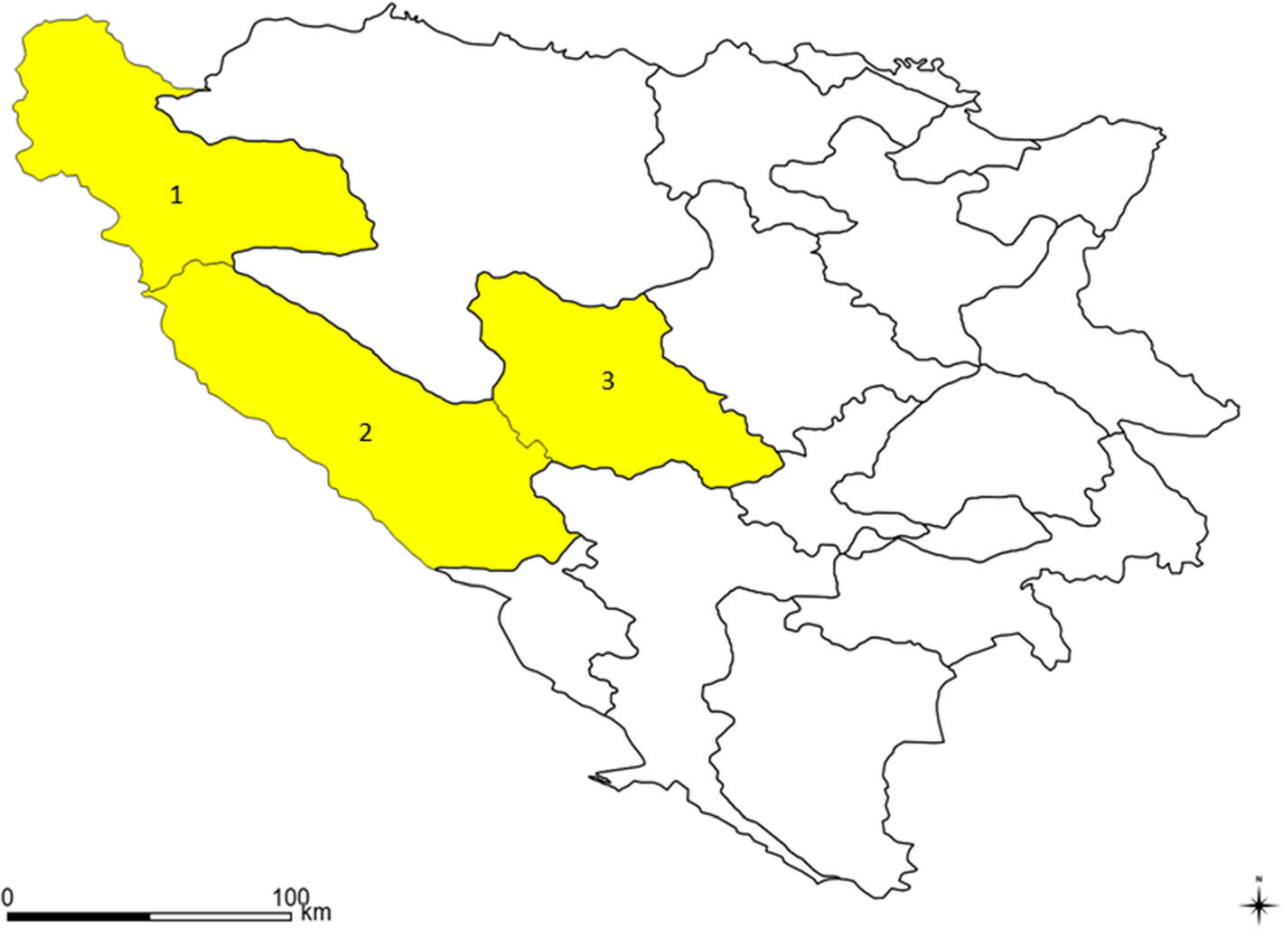
Map of Bosnia and Herzegovina shows the study area consisted of: 1 – Una Sana Canton; 2 – Canton 10; 3 – Central Bosnia Canton

The study was conducted between January and August 2015 in three regions/cantons of the Federation of Bosnia and Herzegovina (FBH). These are Una-Sana Canton with an area of 4125 km^2^, Canton 10 with an area of 4934 km^2^ and Central Bosnia Canton with an area of 3189 km^2^. These cantons represent 23.9% of the area of BH (Fig. 1). In general, the study area is characterised by the temperate continental climate. [25]. This climate in northern and central parts of BH is characterised by four seasons, as well as moderate to warm summers, and mild to moderate winters. Additionally, areas with an altitude higher than 1000 m above sea level are characterised by sub-mountainous and mountainous subtype of the temperate continental climate.

The selection of cantons was guided by convenience for visits, sampling plan as well as the proximity of the veterinary institution where the samples were prepared for testing. The number of dairy cattle in the selected cantons represents 33.9% of the total number of dairy cattle in BH (Table 1) [26].

**Table 1:** Number of cattle in selected regions of BH, given as number of animals and percentage of the total dairy cattle population of BH. [26]

Canton	No. of dairy cows (%)	No. of heifers (%)
Una - Sana	20,881 (18.5%)	3692 (15.2%)
Canton 10	9967 (8.8%)	2145 (8.8%)
Central Bosnia	7460 (6.6%)	1783 (7.4%)
Total	38,308 (33.9%)	7620 (31.4%)

### Study design and sampling of herds

The study was a cross-sectional study that included blood sampling of cattle and interviews of farmers based on a semi-structured questionnaire. The minimum sample size of 60 farms from selected regions of the country (*n* = 180) was determined based upon the detectable herd-level presence of seropositive animals at 5%, 95% level of confidence [27]. The herd sizes were stratified into small (1–10 animals), medium (11–30 animals) and large (> 30 animals). The sample size was slightly increased to 202 herds. From these herds, three were excluded due to missing data, so the final dataset included 199 herds.

### Study animals

The target population was all dairy cattle herds from the selected cantons, which are composed of Simmental, Montafone, Holstein-Friesian, Red Angus breeds and their crosses. The source population was all dairy herds in the selected regions that participated in an annual surveillance and disease control programme focused on bovine brucellosis and enzootic leucosis (“Official Gazette BH”, issue number 34/02). In collaboration with the cantonal veterinary department, a sampling frame of known herds was prepared, and herds were randomly selected based on the available list. The sampling was carried out in a two-level approach, selecting first individual herds and then sampling all animals older than 12 months within each herd. A slightly modified type of sampling was performed in Central Bosnia canton, as animals older than 12 months were previously sampled as a part of the same annual surveillance and disease control program and then herd selection was done randomly based on the available list. The difference between sampling scheme is mainly that the minimum number of animals sampled in each herd was lower in the Central Bosnia Canton. A total of 1970 serum samples were collected from the selected cattle herds. Questionnaire-based interviews were conducted in all farms whose samples are included in this study. In addition to the dairy herds, a small number of beef herds were also sampled.

This study was submitted to and approved by the Ethics Committee of the Veterinary faculty in Sarajevo.

### Sample collection

Blood samples (5 ml) were collected from the tail vein (*v. coccygea*) of each animal, using sterile needles and plain vacutainer tubes. The samples were allowed to stand overnight at room temperature to obtain the serum. Optionally, the samples were centrifuged at 3000 x g for 5 min. Serum was pipetted into cryovials and stored at − 20 °C in the Veterinary Institute of Bihac. Samples were then transported to the Veterinary Faculty in Sarajevo (Department for Infectious Diseases) on ice packs and stored at − 20 °C until tested.

### Laboratory tests

All sera, in their preparation prior to storage, were screened for antibodies against *Brucella* spp. using the Rose Bengal Plate Test (RBPT). Tests were performed according to the standard protocol recommended by the OIE [28]. Positive sera were further tested using the complement fixation test (CFT) as a confirmatory test by standard protocol [28]. Brucellosis positive and negative national control sera were always included during the testing. Complement and hemolysin were obtained from IDEXX/ Porquier, Montpellier, France. Sheep blood was obtained from animals from the farm of the Veterinary Faculty in Sarajevo. The presence of antibodies to *Brucella* was also determined using the IDEXX Chekit Brucellose serum AB test (IDEXX, Switzerland) and interpretation was based on a serum to the positive ratio (S/P%) where < 80% was considered negative and ≥80% positive, according to the manufacturer’s protocol.

The presence of antibodies to *N. caninum* was determined using the IDEXX *Neospora* X2 Ab test kit (IDEXX, Switzerland). A serum with absorbance value (S/P) with a cut-off level of ≥0.50 was considered to be *Neospora* positive. For *C. abortus*, antibody screening was conducted using the IDEXX Chlamydiosis Total Ab test kit (IDEXX, Switzerland). Interpretation of the results was based on S/P% where < 30% was considered negative, ≥30% to < 40% suspect and ≥40% positive. For Q fever (*Coxiella burnetii*) antibody screening was conducted using the IDEEX Q fever antibody test kit and interpretation was based on S/P% where < 30% was considered negative, ≥30% to < 40% suspect and ≥40% positive. Suspect findings for *C. abortus* and Q fever were recorded. The tests were not repeated for suspect results. The test protocol and interpretation of all ELISA tests were performed according to the manufacturer’s instruction (IDEXX). Animals positive to the Rose Bengal Plate Test (RBPT), Complement fixation test (CFT) and Enzyme Linked Immunosorbent Assay (ELISA) test were classified as seropositive to *Brucella.* Animals positive to ELISA tests, according to the manufacturer’s instruction, were classified as being seropositive to *C. abortus*, *C. burnetii* and *N. caninum*. Vaccination against these agents has never been implemented in BH and seropositivity was considered to be due to natural infections.

### Data management and statistical analysis

A database was established in Microsoft Excel® 2013. The raw dataset used in this study is attached in Additional file 1). After cleaning and checking, data were transferred to Stata SE/14 for Windows (Stata Corp., College Station TX) for further statistical analysis. (Questionnaire used in this study please find in the Additional file 2).

Statistical analysis was performed at the individual animal and herd level. The proportion of seropositive animals was estimated using survey data analysis [29], with the herd named as the primary sampling unit and the inverse sampling fraction of the herd as weight. Estimates were also calculated for each canton, age, and breed. The association between seropositivity and these risk factors were calculated using a survey logistic model on individual data, adjusted for the study design. Herd level seroprevalences across cantons were calculated using the simple proportion command in Stata. The overall true individual prevalence was calculated where sensitivity/ specificity of the test was available using the Rogan-Gladen estimator [27].

Finally, a Venn diagram [29] was produced to show the overlap between *C. abortus*, *N. caninum* and *C. burnetii*, and the Goodman and Kruskal’s gamma-statistics was used as a measure of correlation between them.

Data were then collapsed to herd level and herds defined as positive or negative based on test specificity, using the AusVet Epitool. According to the manufacturer’s data, ELISA tests used for investigation of *C. abortus* and *C. burnetii* have demonstrated 100% test specificity (manufacturer’s data). Consequently, herds with at least one reactor / seropositive animal were classified as been infected. However, the ELISA test used for investigation of *N. caninum* has demonstrated a 99.2% test specificity. Taking test properties into consideration, a single reactor was sufficient to classify a herd size up to 7 animals as positive (> 95% herd specificity), two reactors for 7 to 45 animals and three reactors for 45–100 animals. Reactors that have shown positive results on the parallel testing (RBPT + CFT + ELISA) were declared as *Brucella* positive and herds where those reactors were observed were classified as infected.

## Results

In this study, the most frequently detected seropositivity at the individual animal level was against *C. abortus,* with an overall seroprevalence of 52.1%, The seroprevalence of *N. caninum* was 9.2%, for *C. burnetii* 8.8%. The lowest seroprevalence of 0.2% (95% CI: 0.1–0.5) was observed for *Brucella* spp. (data not shown in the table). The *Coxiella burnetii* and *Neospora caninum* tests were assumed to have a sensitivity and specificity close to 1 by the manufacturers. Thus, the estimates did not change substantially when calculating the true prevalence. However, the estimate of the true prevalence of *Chlamydia abortus* was adjusted to 56.6% (95% CI: 44.8–68.2) based on the test properties. Table 2 displays the distribution and individual level seroprevalence across regions, while Table 3 shows the statistical analyses of the same data, adjusted for regional differences.

**Table 2:** Individual animal seroprevalence of *Neospora caninum*, *Chlamydia abortus*, and *Coxiella burnetii* presented over canton, age and breed. (95% CI)

Variables	Category	n=	*Neospora caninum*	*Chlamydia abortus*	*Coxiella burnetii*
All	Total	1970	9.2% (6.0–12.3)	52.1% (41.2–62.7)	8.8% (5.3–14.2)
Canton	Una-Sana	778	11.4% (7.7–16.6)	47.5% (39.5–55.6)	3.2% (2.0–5.2)
	Canton 10	820	8.4% (5.2–13.1)	65.4% (56.6–73.3)	15.3% (10.8–21.2)
	Central-Bosnia	372	8.0% (3.3–18.4)	35.5% (26.2–46.1)	4.3% (1.9–9.3)
Age	Heifers	186	14.8% (9.4–22.5)	62.3% (51.8–71.7)	6.2% (3.2–11.6)
	Cows < 6 years	1725	8.9% (6.2–12.6)	51.4% (39.6–63.0)	8.6% (5.3–13.5)
	Cows > 6 years	59	4.8% (1.4–15.4)	50.8% (37.1–64.3)	22.0% (8.6–45.7)
Breed	Cross breed	483	9.0% (4.0–18.8)	38.7% (27.9–50.7)	5.9% (4.0–8.7)
	Holstein-Friesian	76	12.0% (4.8–26.9)	51.8% (36.5–66.8)	6.8% (2.2–19.5)
	Simmental	1111	9.0% (6.0–13.4)	56.2% (44.5–67.3)	8.3% (3.2–19.6)
	Montafone	138	15.2% (6.9–30.2)	49.5% (37.6–61.4)	15.6% (5.3–38.0)
	Red Angus	162	6.0% (2.2–15.6)	75.9% (60.8–86.5)	18.6% (14.9–23.0)

**Table 3:** Distribution of *Neospora Caninum*, *Chlamydia abortus* and *Coxiella burnetii* in relation to the age and breed using survey logistic regression on individual animal data– adjusted for study design. Results shown as Odds Ratio (95% CI); *p*-value)

Variable	Level	*Neospora caninum*	*Chlamydia abortus*	*Coxiella burnetii*
Age	Heifers	1.00 (−); −	1.00 (−); −	1.00 (−); −
	Cows < 6 years	0.59 (0.33–1.06); *p* = 0.075	0.60 (0.31–1.16); *p* = 0.13	1.47 (0.89–2.42); p = 0.13
	Cows > 6 years	0.29 (0.08–1.08); *p* = 0.065	0.67 (0.32–1.40); *p* = 0.29	4.88 (2.20–10.85); ***p*** **< 0.001**
Breed	Cross breed	1.00 (−); −	1.00 (−); −	1.00 (−); −
	Holstein - Friesian	1.39 (0.36–5.41); *p* = 0.63	1.72 (0.80–3.72); *p* = 0.17	1.20 (0.34–4.24); *p* = 0.78
	Simmental	0.99 (0.41–2.41); *p* = 0.99	2.02 (1.14–3.58); **p = 0.016**	1.44 (0.63–3.26); *p* = 0.39
	Montafone	1.74 (0.52–5.83); *p* = 0.37	1.49 (0.77–2.86); **p = 0.029**	2.99 (0.85–10.51); **p = 0.09**
	Red Angus	0.65 (0.16–2.59); *p* = 0.54	5.05 (2.10–12.18); **p < 0.001**	3.82 (2.27–6.44); ***p*** **< 0.001**

**Fig. 2:**
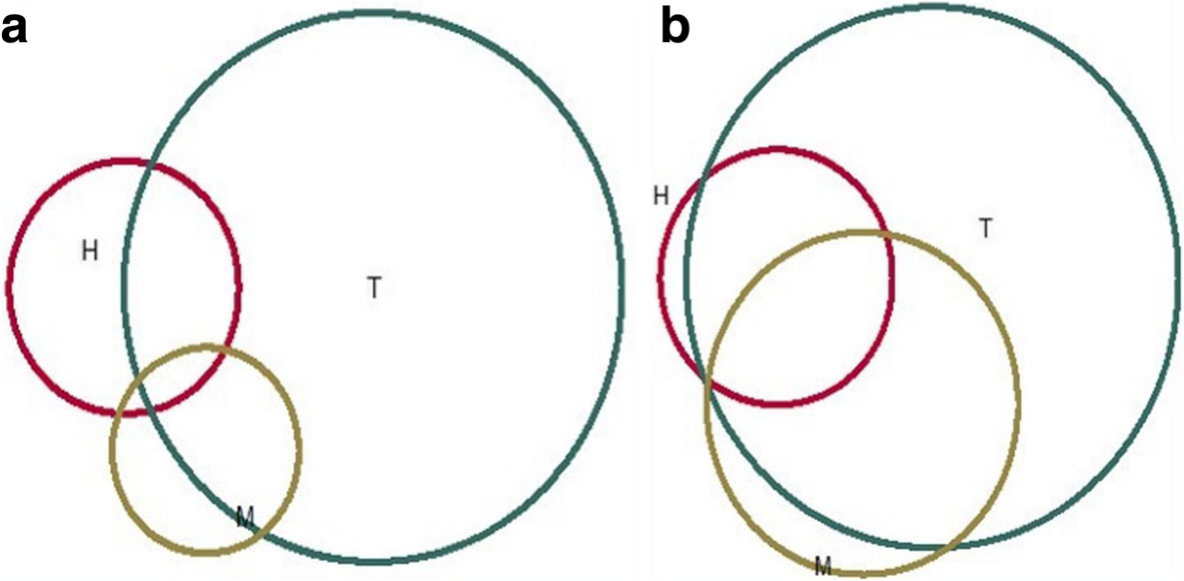
Observed overlap in seroprevalences of *Neospora caninum*, *Coxiella burnetii* and *Chlamydia abortus* at individual animal level (**a**) and herd level data (**b**). H – *Neospora caninum*; M – *Coxiella burnetii*; T – *Chlamydia abortu*s; Blue square represents an investigated population

Cows older than six years were associated with a higher seropositivity for *C. burnetii*. Further, there were breed predispositions in Red Angus (OR 5.05; *p* < 0.001), Simmental (OR 2.02; *p* = 0.016) and Montafone (OR 1.49; *p* = 0.029) for *C. abortus* compared to cross breed. In addition, there were breed predispositions in Red Angus (OR 3.82; p < 0.001) and Montafone (OR 2.99; *p* = 0.09) for *C. burnetii* compared to cross breed. There was a substantial number of multi-seropositive individuals for *N. caninum*, *C. abortus* and *C. burnetii* (Fig. 2a). The observed overlap was, however random for *N. caninum* vs. *C. abortus* and *C. burnetii*, (gamma close to 0) while a higher correlation was found for *C. abortus* and *C. burnetii* (gamma = 0.35)*.*

Table 4 shows the results for the herd level data. Regional differences at the herd level were found, except for *N. caninum*. Most herds in all three cantons were seropositive for *C. abortus*. In addition, the proportion of seropositive herds in Central Bosnia Canton (4.1%) was notably different in comparison with Una-Sana Canton (27.7%) and Canton 10 (45.9%), for *C. burnetii*. Regional differences at the herd level were found using chi-square test (*p* < 0.001). A total of 4.6% (95% CI: 1.5–13.5) *Brucella* spp. reactors positive herds were found, but only in Una-Sana Canton (data not shown in the table).

**Table 4:** Overall and cantonal proportions (95% CI) of *Neospora caninum*, *Chlamydia abortus*, and *Coxiella burnetii* seropositive herds (*n* = 199)

Canton	Herds	*Neospora caninum*	*Chlamydia abortus*	*Coxiella burnetii*
Una-Sana	65	40.0% (28.7–52.4)	96.9% (88.3–99.2)	27.7% (18.1–39.9)
Canton 10	37	40.5% (25.9–57.1)	97.3% (82.6–99.6)	45.9% (30.5–62.2)
Central-Bosnia	97	29.9% (21.6–39.8)	78.4% (68.9–85.5)	4.1% (1.5–10.6)
Total	199	35.2% (28.8–42.1)	87.9% (82.6–91.8)	19.6% (14.6–25.8)

Figure 2b shows the herd-level overlap for *N. caninum*, *C. abortus* and *C. burnetii* using the Venn diagram. As for individual data, the observed overlap was more random for *N. caninum*, but a higher correlation (gamma = 0.73) was found for *C. abortus* and *C. burnetii.*

## Discussion

This study demonstrates that cattle in three regions of BH were frequently seropositive to *C. abortus*, less frequently to *C. burnetii* and *N. caninum* and rarely to *Brucella* spp. Recent studies on the seroprevalence of *C. abortus* in sheep in BH reported an overall seroprevalence of 43.3% at the individual and 84.2% at the herd level [30]. In addition, *Chlamydia* infection in goats has been previously reported in the southern part of BH [31]. Our study represents the first insight into the presence of *C. abortus* infection of cattle in BH. Moreover, several studies have reported substantial variation in the seroprevalence of *C. abortus* infection in cattle worldwide. In a study on cattle from Turkey [32], a *C. abortus* seroprevalence of 8.3% was reported at the individual animal level, in cows with histories of abortion and 26.9% at the herd level. In Poland, a total of 19.3% out of 1333 bovine sera tested positive for *C. abortus* and *C. psittaci* [33]. Generally, anti*-C. abortus* antibodies were found more frequently in our study than in the above-mentioned studies. The seroprevalence of *C. abortus* did not vary significantly across the regions and age groups. Contrary to our finding, a study from Jordan reported significant regional differences in addition to differences between age groups [34]. In the current study, Red Angus was the breed with the highest *C. abortus* seroprevalence noted. This might be explained by the differences in farm management between dairy and beef cattle in BH. Red Angus cows were kept for several months in the overpopulated stables, which increased the contact between animals and exposing animals to contaminated feed and the infectious environments. The seroprevalence of *C. abortus* may be overestimated in terms of antigenic cross-reactivity between *Chlamydia* species, which may also operate as co-infections in the same herd or in the same animal [35]. Therefore, planned follow-up studies using molecular tests will presumably improve our knowledge regarding chlamydial infection in cattle. The vaccination control programme against *C. abortus* infection in cattle are not currently being implemented in BH. Also, *C. abortus* is not listed as a causative agent in the annually updated reporting strategy for severe reproductive failures in cattle (“Official Gazette of BH” issue number 4/16). Moreover, our field experiences have shown that the implementation of biosecurity measures are not common practice among farmers. Hence, the high level of antibodies to *C. abortus* found in this study, indicates the need for further epidemiological investigations.

In this study, the overall seroprevalences of *N. caninum* on individual and herd level were 9.2% and 35.2%, respectively. The occurrence was widespread, with no evident difference between regions, age groups, and animal breeds. A recent study [20] reported the presence of anti-*N. caninum* antibodies in ruminants in BH, with 16 (8.7%) of 184 positive samples in the period of 2005–2009. In Croatia, the seroprevalence of *N. caninum* antibodies was reported at 5.8% [36]. In Serbia, it was reported [37] that individual and herd seroprevalences were 4.6% and 27%, respectively. Another study from the northern part of Serbia showed an overall individual seroprevalence of 15.4% using ELISA and indirect fluorescent antibody test (IFAT) [38]. The difference of estimates between our study and studies from neighboring countries may be explained by the differences in study designs, the diagnostic test used, sample size and management related factors. The overall picture, however, is that *N. caninum* is frequently found and represents a potential cause of reproductive failures in BH, thus being subject to future attention in Balkan countries.

Q fever is not a part of the Directive for control of infectious animal diseases in BH (“Official Gazette” No 34/02) and information about this pathogen in cattle populations is very scarce on a national level. Previous studies reported the occurrence and spread of Q fever in the human populations of BH [39, 40]. Our study found an overall seroprevalence for *C. burnetii* at the individual and herd level of 8.8% and 19.6%, respectively. A recent study from Croatia [41] reported that 2.7% of cattle tissue samples were positive for the presence of *C. burnetii* DNA. In the same study, 13 novel *C. burnetii* genotypes unique for Croatia were reported. In Albania, the reported seroprevalence [42] of Q fever was 7.9% in cattle. The circulation of *C. burnetii* among cattle population indicates the need for its control in BH.

The overall individual seroprevalence of *Brucella* spp. in cattle in our study was 0.2%. In 2008, while a test-and-slaughter control strategy was still being used in cattle and small ruminants, a seroprevalence of 4.6% was found, mainly in sheep and brucellosis in humans was also reported [43]. Authorities in BH then changed the control strategy, and mass vaccination of small ruminants with the ocular Rev-1 vaccine was implemented in 2009. Importantly, as reported in the last FAO regional workshop on brucellosis control in Central Asia and Eastern Europe, the prevalence of brucellosis in cattle has been reduced in BH, due to the vaccination strategy [21]. Our study validates this trend and underlines that anti-*Brucella* antibodies detected in cattle in BH could be possible due to a spill-over of *B. melitensis* from the small ruminant reservoir. Although proving the absence of *B. abortus* is impossible, cattle being the spill-over host of *B. melitensis* originating in the small ruminant reservoir could be considered as a possible explanation for the low prevalence of anti-Brucella antibodies detected in cattle. It is important to re-emphasize that the favorable situation in cattle follows the implementation of a successful vaccination program in small ruminants in BH, as it has been suggested in different countries in the region [21]. Such findings suggest that in mixed small ruminants-cattle herds, a successful vaccination program in small ruminants may result in controlling *B. melitensis* spill-over infection to cattle without the need to vaccinate cattle [44, 45].

Our results indicate that *N. caninum, C. abortus,* and *C. burnetii* are frequently present in cattle herds in BH. This may be of clinical importance for reproductive disorders and may restrain the production performance of cattle herds. Importantly, brucellosis in cattle resulting from the spill-over of *B. melitensis* is most probably controlled in BH as a result of implementation of a successful mass vaccination program in small ruminants. The very few positive animals, found in this study could be possible linked to a spill-over of *B. melitensis* from sheep and goats. Currently, brucellosis is not contributing significantly to reproductive disorders in cattle in BH. Today, small ruminants are not tested, as they are under vaccination program. There is an annual program for brucellosis in cattle. A rise in the prevalence of anti-*Brucella* antibodies in cattle may indicate a problem in the control program in small ruminants. *N. caninum* is frequently found in cattle herds of BH which may be, probably linked to an un-controlled population of dogs. For *C. burnetii* and *C. abortus,* the high levels found in this study call for attention as possible constraints to the cattle reproduction and breeding in BH. Continued studies will investigate the potential impact of these above-mentioned agents on reproductive performance in cattle in BH. Also, we found a high correlation (gamma = 0.73) between *C. abortus* and *C. burnetii*, while the observed overlap between other agents were more random. This may be partially explained as the existence of common factors that contribute to the occurrence of these agents. Nevertheless, this association should be investigated further.

## Conclusions

The study demonstrates a high level of antibodies to *Chlamydia abortus* in BH cattle herds, but substantial levels of antibodies to *Coxiella burnetii* and *Nesopora caninum* were also found. The findings illustrate a situation where these agents may be influencing reproductive performance in the cattle population. Currently, *Brucella* spp. does not seem to represent a reproductive problem in cattle in the studied regions.

## Additional files

Additional file 1: Softic_BMC_raw_dataset; The raw dataset used in this study. (XLSX 147 kb)

Additional file 2: Softic_BMC_questionnaire; The questionnaire used in this study. (DOC 102 kb)

## Abbreviations

BH: Bosnia and Herzegovina
BVDV: Bovine viral diarrhoea virus
ELISA: Enzyme linked immunosorbent assay
FBH: Federation of Bosnia and Herzegovina
OR: Odds ratio
