# Supplementary material for: The serostatus of Brucella spp., Chlamydia abortus, Coxiella burnetii and Neospora caninum in cattle in three cantons in Bosnia and Herzegovina
Source: BMC Vet Res. 2018 Feb 2;14:40. doi: 10.1186/s12917-018-1361-z (PMC5797338; doi:10.1186/s12917-018-1361-z)
Supplement: Supplementary file 2 — Softic_BMC_questionnaire; The questionnaire used in this study. (DOC 102 kb) [file 12917_2018_1361_MOESM2_ESM.doc]

**Questionnaire** – Risk factors connected with reproductive disorders in cattle in Bosnia and Herzegovina (USK, C10, and SBK)

Investigator: ___________________________ Date: ________

**Farmer / Owner information**

Gender M / F Age: Producer ID: ___________________________

Occupation: _________________________

**Farm level information**

Region_________ Town___________ Local community_______________

Farm owner: Private Public

Distance of farmer’s house from main facilities? ______________________

Number of workers on the farm? _______________________________________________

New workers on the farm? Yes / No;

If yes, seasonally__ or permanently__ employed? (Mark)

Technical assistance, if any? ___________________________________________________

Farm ID: ______________________ Farm type: ____________ Herd size (Total number): __________

Cows: _____; Heifers: _____; Bulls_____; Calves_____; Dead calves_____; Dehorned calves_____;

Number of sampled animals (blood samples) ______________

Number of milk samples from dairy farm ______________

1. Type of breeds in the farm?

Holstein-Frisian Simmental Cross breeds

Others/Specify: _______________________

2. Is there any other species of farm animal sharing facilities with cattle? Yes / No

If yes, which species (mark in the corresponding cell how many of them?)

| Type of farm animal | |
| --- | --- |
| Equine |  |
| Sheep & Goats |  |
| Dogs |  |
| Cats |  |

Are there any other species of companion or wild animals appearing on the farm? Yes / No

If yes, which species (mark in the corresponding cell how many of them)

| Type of animal | Number |
| --- | --- |
| Wild birds in barn |  |
| Rodents |  |
| Hedgehogs |  |
| Foxes |  |
| Others |  |

3. Does this herd have any contact with another herd? Yes / No

Does this farm have any contact with another farms and animals from another farm? Yes / No

4. Type of housing? Tie stall______ Free stall______ Combination of tie stall and free stall______

- Tie stall
- Area__________ m^2^
- Floor type: Wood___ Concrete__ Soil__
- Area where the cows lay__________m^2^
- Floor type: Alley – Wood__ Concrete__ Soil__

Laying area – Wood__ Concrete__ Soil__ Straw__ Saw dust__ (Mark)

- Is there bedding in lying area? Yes / No
- Does the stall have drainage? Yes / No
- Does the stall have appropriate ventilation? Yes / No
- Temperature (Volcraft device 4 in 1 - ◦C) __________
- Intensity of light (Volcraft device 4 in 1 – lux) __________
- Relative humidity (Volcraft device 4 in 1 – %)___________
- Noise in stall (Volcraft device 4 in 1 – dB) __________
- Hygienic status of the farm? Mark according to cleanliness category (**1**-**5**) ____

Remark: Cleanliness = http://www.ansci.umn.edu/prod/groups/cfans/@pub/@cfans/@ansci/documents/asset/ansci-dairydiag-wkshts-13.pdf

5. Does the farm have separate calving pen? Yes / No

If yes; Area______m^2^; Floor type ___________

How often is this area cleaned? _____________

Have you ever had the appearance of breeding diseases? Yes / No

If yes, which disease(s): ________________________________________________________

6. Does the farm have calf pen? Yes / No

Individually / Groups; How much calves per group? ______;

Area: Total_____m^2^; Per animal_____m^2^;

Laying area – Wood__ Concrete__ Soil__ Straw__ Saw dust__ (Mark)

7. Husbandry:

- Type of insemination? AI____ Bull____ Both____
- Source of replacement cattle? Own____ Purchase____ Both____

Purchase animals in last 12 months ________; From how may units? ________

Last purchase animals: ___________________________________________________

- Main reasons of culling animals over the last five years:

________________________________________________________________________________________________________________________________________________

8. Method of waste disposal? (Mark in corresponding cell)

| Type of waste | Method of disposal | | |  |
| --- | --- | --- | --- | --- |
|  | Feeding dogs | Burial | Waste dumps | Other |
| Aborted material |  |  |  |  |
| Offal |  |  |  |  |
| Dead animals |  |  |  |  |

9. The main type of cattle feeding

In case with feeding with different food, mark according to frequency: **1**-rarest, **5**-usually

- Crop residues______
- Industrial by-products (Wheat and corn bran, molasses, oil seed cake and others) ______
- Pasture______
- Hay made of local pasture______
- Silage______
- Other / Specify________________________________________________________

10. Grazing pattern: Zero grazing____ Partial grazing____ Free range & supplements____

11. What is your water source?

- Public______
- Open water source (Pond, River, Spring water, Well or Rainwater) ______

12. Is there any risk of feed contamination with dog faeces? Yes / No

13. Are there dogs in the farm? Yes / No; If yes, how many? ____

14. How long have you had dog/s in the farm? ____

15. Have you ever dewormed or vaccinated your dog/s? Yes / No

- If yes, name the diseases for which your dog/s were vaccinated or treated

Treatment_______________ Vaccination________________

16. Do the dogs access the cows feed store? Yes / No

17. Do you dispose the faeces of your dog/s? Yes / No

18. How do you dispose dog/s faeces? Waste dump____ Burial____ Cesspit____Other____

19. What is the major feeding for your dogs? ____________________________________

20. Do you have veterinary service available? Yes / No

- If yes, Public____ Private____ Both____

21. What are major production constrains facing your farm? (Mark in corresponding cell, according to frequency; **1**-never; **5**-very often)

| Production constrains | |
| --- | --- |
| Feed |  |
| Diseases |  |
| Replacment stack |  |
| Market |  |
| Veterinary service |  |
| AI service |  |
| Space |  |
| Waste disposal site |  |
| Others |  |

Disease constrains information:

Type of disease: ____________________________________________________

Udder diseases: ___; Reproduction failures: ___; Digestive disorders: ___; Others: ___

Reccurence and frequency of diseases (Mark in corresponding places; **1**-never; **5**-very often)

| **Udder diseases** |  |
| --- | --- |
| Agalactia |  |
| Supernumerous teats |  |
| Severe clinical mastitis |  |
| Mild clinical mastitis |  |
| Teat injury |  |
| Papillomas/neoplasma |  |
| Udder oedema |  |
| Drying off treatments |  |

| **Digestive diseases** |  |
| --- | --- |
| Indigestion |  |
| Colic |  |
| Bloat |  |
| Dental disorders |  |
| Parasitic disease |  |
| Liver disease |  |
| Contagious Diarrhoea |  |

| **Other** |  |
| --- | --- |
| Listeriosis |  |
| Pasteurellosis |  |
| Catharral fever |  |
| Tick borne diseases |  |
| Clostridial diseases |  |
| Claw diseases |  |

22. Is there any access of wild animals to your farm premises? Yes / No

Individual level information

| Animal No | Breed | Age (M) | Age @ 1st AI | Av.no Serv. | Origin | No. of pregnancy | No. Of calvings | Open days (M) | Calving Intervall (M) | Reproductive Disorder | Type of RD | Term of RD | Frequency | Terminated Pregnancy | Maternal RD (Yes/No) |
| --- | --- | --- | --- | --- | --- | --- | --- | --- | --- | --- | --- | --- | --- | --- | --- |
|  |  |  |  |  |  |  |  |  |  |  |  |  |  |  |  |
|  |  |  |  |  |  |  |  |  |  |  |  |  |  |  |  |
|  |  |  |  |  |  |  |  |  |  |  |  |  |  |  |  |
|  |  |  |  |  |  |  |  |  |  |  |  |  |  |  |  |

***RD:*** *Reproductive disorder (Yes/No),* ***Origin****: Purchased or home breed,* ***M:*** *months,* ***AI****- artificial insemination; Maternal reproductive disorder (Yes/No/NA), Type of reproductive disorder: Acronyms:* ***AB****-abortion;* ***MT****-metritis;* ***RFM****-retention of placenta;* ***CDC****-congenital defective calf;* ***RB****-repeat breeder;* ***SB****-stillbirth;* ***UP****-uterus prolapse;* ***UT****- uterus torsion;* ***PG****- prolonged gestation;* ***DY****- dystocia;* ***AO****- anoestrus;* ***SO****- silent oestrus;* ***OC****- ovarian cysts*

Milk information:

Average milk yield per cow (L/Kg in 305 days of lactation):

Primiparous: _____

2^nd^ lactation: _____

3^rd^ lactation: _____

Milk fat value (%) _____

Milk protein value (%) _____

Bacterial count (per mL) _____

Personal observation:_____________________________________________________________________________________________________________________________________________________________________________________________________________________________________________________________________________________________________________________________________________________________________________

Thank you very much!
